# PReS-FINAL-2130: Antibodies to MDA5 correlate with a distinct phenotype in children with juvenile dermatomyositis, including higher risk of lung involvement and ulcerative skin disease

**DOI:** 10.1186/1546-0096-11-S2-P142

**Published:** 2013-12-05

**Authors:** E Moraitis, S Tansley, K Arnold, ZE Betteridge, H Gunawardena, TS Jacques, C Owens, LR Wedderburn, N Mchugh

**Affiliations:** 1 Rheumatology Department, Great Ormond Street Hospital for Children, London, UK; 2 Rheumatology Department, Royal National Hospital for Rheumatic Diseases, Bath, UK; 3 Rheumatology Unit, UCL Institute of Child Health, London, UK; 4 Bath Institute for Rheumatic Diseases, Bath, UK; 5 Rheumatology Department, North Bristol Hospital, Bristol, UK; 6 Department of Histopathology, Great Ormond Street Hospital for Children, London, UK; 7 Neural Development Unit, UCL Institute of Child Health, London, UK; 8 Radiology Department, Great Ormond Street Hospital for Children, London, UK

## Introduction

Myositis specific autoantibodies (MSA), exclusively found in patients with Idiopathic Inflammatory Myopathies can be detected in approximately 60% of children with JDM. Anti-MDA5 antibodies, a subgroup of MSA, appear to be associated with clinically amyopathic myositis, rapidly progressive interstitial lung disease (RP-ILD) and a poor prognosis in adult East Asian dermatomyositis patients. Small studies in Japanese children with JDM have suggested similar disease phenotype. This contrasts dramatically with findings in predominantly Caucasian US adult populations where anti-MDA5 has been associated with a distinct cutaneous phenotype and no association with RP-ILD has been found.

## Objectives

We aimed to determine the frequency and associated clinical phenotype of anti-MDA5 autoantibodies in a large UK based, predominantly Caucasian, cohort of patients with JDM.

## Methods

Serum samples, from 285 patients with JDM were obtained through JDM National (UK and Ireland) Cohort and Biomarker Study and Repository for Idiopathic Inflammatory Myopathies. The presence of anti-MDA5 antibodies was determined by ELISA using recombinant MDA5 protein. Results were compared with matched clinical data, muscle biopsies (scored by a single experienced paediatric neuropathologist) and chest imaging (reviewed by a single experienced paediatric radiologist). Both biopsy scoring and imaging review were performed blind to clinical or serological data.

## Results

Anti-MDA5 antibodies were identified in 7.4% of JDM patients and were associated with a distinct clinical phenotype including skin ulceration (p = 0.025), oral ulceration (p = 0.013), arthritis (p = 0.002) and milder muscle disease both clinically (as determined by a higher lowest ever Childhood Myositis Activity Score (p = 0.024)) and histologically (as determined by a lower JDM muscle biopsy score). Five out of 14 children had radiological evidence of interstitial lung disease (ILD) but none had RP-ILD. Despite these associations children with anti-MDA5 had an improved prognosis and a greater proportion achieved disease remission at 2 years post diagnosis according to PRINTO criteria (p = 0.023).

## Conclusion

Anti-MDA5 antibodies can be identified in a small but significant proportion of UK children with JDM. They identify a distinctive subgroup with an increased risk of skin and oral ulceration, arthritis, ILD and milder muscle disease, both clinically and histologically. Screening for anti-MDA5 autoantibodies at diagnosis would be useful to guide further investigation for possible lung disease, inform on prognosis and potentially to confirm the diagnosis, as subtle biopsy changes could otherwise be missed.

## Disclosure of interest

None declared.

